# Robust perceptual-load-dependent audiovisual integration in adult ADHD

**DOI:** 10.1007/s00406-022-01401-z

**Published:** 2022-04-05

**Authors:** Marcel Schulze, Behrem Aslan, Paul Jung, Silke Lux, Alexandra Philipsen

**Affiliations:** 1 Department of Psychiatry and Psychotherapy, University of Bonn, 53127 Bonn, Germany; 2 Faculty of Psychology and Sports Science, Bielefeld University, Bielefeld, Germany

**Keywords:** Attention-deficit/hyperactivity disorder, Multisensory integration, Perceptual decision-making

## Abstract

We perceive our daily-life surrounded by different senses (e.g., visual, and auditory). For a coherent percept, our brain binds those multiple streams of sensory stimulations, i.e., multisensory integration (MI). Dependent on stimulus complexity, early MI is triggered by bottom–up or late via top–down attentional deployment. Adult attention-deficit/hyperactivity disorder (ADHD) is associated with successful bottom–up MI and deficient top–down MI. In the current study, we investigated the robustness of the bottom–up MI by adding additional task demand varying the perceptual load. We hypothesized diminished bottom–up MI for high perceptual load for patients with ADHD. 18 adult patients with ADHD and 18 age- and gender-matched healthy controls participated in this study. In the visual search paradigm, a target letter was surrounded by uniform distractors (low load) or by different letters (high load). Additionally, either unimodal (visual flash, auditory beep) or multimodal (audiovisual) flanked the visual search. Linear-mixed modeling was used to investigate the influence of load on reaction times. Further, the race model inequality was calculated. Patients with ADHD showed a similar degree of MI performance like healthy controls, irrespective of perceptual load manipulation. ADHD patients violated the race model for the low load but not for the high-load condition. There seems to be robust bottom–up MI independent of perceptual load in ADHD patients. However, the sensory accumulation might be altered when attentional demands are high.

## Introduction

Attention-deficit/hyperactivity disorder (ADHD) is a neurodevelopmental disorder accompanied with varying levels of inattention, hyperactivity and/or impulsivity [1]. About 40% to 50% of people show symptoms beyond the childhood, raising the scientific focus to adult ADHD in the past recent years [2]. Besides the core symptoms that are highly linked to executive functioning [3], ADHD patients also show anomalies in basal sensory processing, although this topic is rather under researched. Sensory processing in ADHD is mainly marked by deficits in early stimulus-modulatory processes (e.g., inhibition of irrelevant stimulus characteristics or distractors) especially in the auditory domain, but also reported for the visual sense, touch, taste, smell [4]. Overall, patients with ADHD can be described as being sensory hypersensitive [5, 6].

Despite the interplay of the core symptoms of ADHD and sensory processing are not fully understood yet, the difficulties in sensory processing seem to extend in adulthood [5]. Former studies most frequently investigated single sensory domains. However, the complexity of the environment and our daily-life interaction with it demands to process multiple sensory streams in parallel. By combining different stimulus streams to reach a unified percept, a process that is called multisensory integration (MI), our brain is optimally adapted to detect, locate and differentiate external objects, compared when confronted with only one unimodal stream [7]. To integrate successfully, attention seems to play a major role in MI, whereby the exact role of attention as a condition for MI is subject of an ongoing debate [8]. Depending on stimulus complexity, MI can occur at early, pre-attentive stages (bottom–up triggered MI) for simple stimuli or at later stages, i.e., top–down attentional effects for more complex stimuli (e.g., semantic stimuli) [8, 9]. With its attentional difficulties and abnormalities in unimodal sensory processing, ADHD is a valuable clinical condition to study MI, raising the intriguing question whether ADHD patients show deficits in MI, in dependence of varying attentional demands. Only a few studies investigated MI in adult ADHD, yielding mixed results. In one study, ADHD patients did not improve speech-in-noise understanding to a similar degree like healthy controls, when the visual source, namely the lip movements of the speakers were added. In other words, there were no beneficial effects from a multimodal source in ADHD patients [10]. Such a multimodal benefit, measured in an acceleration in the detection of multimodal stimuli compared to unimodal stimuli, was evident in McCracken et al. [11]. They mixed simple stimuli (an auditory beep) with complex stimuli (the spoken word ‘red’), instead of using only complex stimuli like speech. A recent study investigated MI using simple stimuli (namely the sound-induced flash illusion, where simple visual flashes and auditory beeps were used) and complex stimuli (namely the McGurk illusion where speech-like phonemes are used) in an adult ADHD sample compared to healthy controls [12]. Here, ADHD patients showed intact MI abilities for simple stimuli but failed to integrate the complex stimuli. While the bottom–up MI seems to be intact, the top–down MI in ADHD patients seems to be deficient.

In everyday life, not only the attentional demands, but also in addition the perceptual demands can be very different. The model of perceptual capacity was suggested in Lavie’s perceptual load theory to be limited but this capacity is always involuntarily filled. Selective attention, i.e., the ability to attend to one source of information while ignoring others [13, 14] is therefore dependent on the available perceptual capacity.

When confronted with a task composed of a high load, the perceptual capacities are reached resulting in an early selection, hence no distractors or task-irrelevant stimuli can be processed, contrary, in a low-load task where free perceptual resources allow for additional processing of distractors due to a late perceptual selection [15]. Studies combining perceptual load and MI in healthy subjects yield mixed effects but there seem to be an ‘immunity-effect’ of MI for perceptual load [16–18]. In other words, reaction time benefits for the detection of multimodal stimuli, as compared to unimodal stimuli presentation, seem to be robust irrespective of the perceptual load. In ADHD, studies investigating MI in dependence of the given perceptual load are missing. In paradigms, where the perceptual load was manipulated (without measuring sensory processing or MI), ADHD patients showed less distraction for the higher load condition [19, 20]. The current study challenges the robustness of bottom–up MI in ADHD by manipulating simple stimuli MI through additional task demands. This manipulation is realized by varying the attentional capture through perceptual loads.

By combining bottom–up stimuli with varying amounts of perceptual capacities to process those stimuli, we assume, that ADHD-patients’ limits in MI are reached for the high load, since they are more focused and less distracted by the sensory stimuli. In other words, the immunity effect for load-dependent MI should not hold for ADHD patients since once their perceptual capacities are reached in the high-load conditions, the reaction time benefit for multimodal stimuli should be diminished.

## Materials and methods

### Participants

Eighteen adult ADHD patients [7 females, age: 34.7 (SD: 9.3) years] were recruited at our psychiatric outpatient department and compared to 18 healthy controls [7 females; age: 35.6 (SD: 11.8) years], recruited through bulletin boards.

According to international and German guidelines [21, 22], patients were diagnosed by a specialist psychiatrist following a detailed clinical and psychosocial interview that integrates somatic differential diagnosis, the patients’ psychiatric and developmental history, and observer reports.

ADHD symptoms were further assessed with the Conners Adult ADHD Rating Scale (CAARS-long version self-rated) [23] and the Wender Utah Rating Scale (WURS-k) for ADHD childhood symptoms [24]. Borderline personality disorder, as a frequently associated comorbidity was screened with the borderline symptom list (BSL-23) [25].

In addition, depressive symptoms were self-rated with the Becks Depression inventory [26]. In case of medication intake (e.g., methylphenidate), ADHD patients were asked to discontinue at least 24 h prior to the experiment. Adult sensory profile (ASP) was administered to measure sensory processing according to Dunns’ model, i.e. person being sensory seeking, sensory avoidant, sensory sensitive or low registering to sensory stimuli [27].

All participants gave written consent to take part, and the study was approved by the ethics committee of the medical faculty of the University of Bonn (408/20).

### Behavioral experiment

The experiment took place in a light-dimmed room and participants were seated at a distance of approximately 60 cm in front of a screen. Auditory stimuli were presented via headphones (Sennheiser HD300 pro) with adjusted sound pressure level for each participant.

### Paradigm

Paradigm was adapted by Lunn et al. [16] and programmed with Presentation software (Version 22.1, Neurobehavioural Systems, http://www.neurobs.com). Each trial began with a central fixation cross (presented for 500 ms) followed by the load condition, the sensory condition or the control condition. In the load condition, the participants’ task was to press a button as fast as possible when they perceived an X on the screen. The target letter was, in case of the high-load condition, embedded among six circularly arranged (2.0^o^ radius) distractor letters (H, K, M, V, W, Z) and in the low-load condition embedded among placeholders (O’s). The position of the target letter was randomized across trials and total presentation time was 200 ms, with 4 load blocks (each comprised of 40 trails) for low- and high-load respectively. The sensory conditions comprised peripheral targets presented either left or right from the circular array of letters and the participants should indicate by button press, whether the event occurred left or right. Of note, in those trials, no target letter was present, but the distractors were visible according to the low/high-load condition. The sensory stimuli comprised a short auditory beep tone (1KhZ; 100 ms) and a short visual flash presented unimodal (auditory or visual alone) or multimodal (parallel presentation of auditory and visual stimuli). In total, 18 sensory events were presented interleaved for each load (with target letter) condition. As a control condition, only distractors were presented hence neither an ‘X’ nor an event of the sensory trial was presented (22 trials in within a block). Here, participants should not react at all. The participants completed four blocks per load with an interleaved design counter-balanced across subjects (either ABBAABBA or BAABBAAB) resulting in total of 640 trials. Prior to the main experiment, a training took place entailing all conditions (~ 5 min) to make the participants familiar with the tasks.

### Statistical analysis

All demographical variables were group-wise compared with independent samples *t* tests.

For all conditions, reaction times were outlier corrected with the MATLAB build in function ‘rmoutliers’ i.e., defining an outlier with a value above 3 median absolute deviation (MAD). Reaction time data for the unimodal and multimodal conditions were then fitted to one linear mixed model (LMM) using the package lme4 implemented in R [28]. LMMs are advantageous over classical variance analysis approaches since they also account for single observations for each subject in a given condition, rather than averaging the condition/subject [29]. With reaction time as a dependent variable, model comparisons were realized with Akaike information criterion (AIC). Here, the model with fixed effects of group, loading and modality (uni-, multimodal) and random effects of subject and trial order lead to the lowest AIC. Variance components were estimated with restricted-maximum likelihood (REML). Type II Wald Chi-square tests were applied to derive *p* values from interactions. To further inspect the influence of group, loading and conditions on RT, least-squared means were extracted and compared in a contrast analysis with Bonferroni p value adjustments.

Percentage of right answers, as well as commission and omission errors of the sensory conditions were averaged per condition and compared by 2 × 2 × 2 two-way repeated measurements analysis of variance (ANOVA) with group, modality, and loading as factors. Similarly, comparisons of the reaction times and errors of the control condition were entered in separate ANOVA’s as described above.

#### Modeling multisensory reaction time acceleration

Presenting multimodal cues can lead to a faster detection in terms of reaction time compared to uni-sensory presentation only. The, so-called redundant signal effect leads to perceptual decision-making by statistical facilitation where the faster processed uni-sensory cue triggers a motor response [30, 31]. Of note, this is opposed to ‘pooled’ sensory processing models where an interaction between the uni-sensory processing modalities reaches a threshold enabling a motor response. To address MI processing as a function of statistical facilitation, the race model inequality was calculated [32]:

$${P}_{AV}(t)\le {P}_{A}\left(t\right)+{P}_{V}(t)$$

where *P* representing the cumulative probability-motor response at a time *t* for the respective conditions denoted with *A* auditory only, *V* visual only, AV audiovisual. The race model assumes that no interactions between uni-sensory processing units occur, hence the sum of the cumulative distribution function of the reaction time in AV condition may not exceed the sum of the cumulative distribution for the two uni-sensory processing units. The race model was implemented in MATLAB using the RSE toolbox [33]. As a first step, individual cumulative distribution functions were calculated for each condition per load. Next, the Miller’s bound was calculated i.e., the upper bound for assuming statistical independence of the two uni-sensory processing units. Violation of the race model was calculated by examining the size of the area between Miller’s bound and the distribution of the MI distribution function [34] and statistical assessed by one-sample *t* tests for each load within the groups. The upper (race model violation) and lower (multisensory benefit) bounds were group-wise compared with a two-sample *t* test.

## Results

### Demographics

For an overview of demographical variables, see Table 1. There was no significant group difference in terms of age, and gender. ASP were significantly different between groups for low registration (*t*(35) = − 5.07, *p* < 0.001), sensory avoidance (*t*(35) = − 2.48, *p* = 0.002), sensory sensitivity (*t*(35) = − 3.59, *p* = 0.001) but not for sensation seeking (*t*(35) = − 0.287, *p* = 0.77).

**Table 1:** Demographical description

	ADHD (*n* = 18; 7 f)	Controls (*n* = 18; 7 f)
Age (SD)	34.7 (9.33)	35.6 (11.8)
CAARS—inattention**	19.17	4.19
CAARS—Hyp./Imp.**	14.44 (4.4)	2.94 (2.54)
Wurs-k **	36.59 (15.3)	11.62 (12.6)
BDI*	10 (9.8)	2.3 (4.6)
BSL-23	14.9 (15.7)	4.4 (6.9)
Comorbidities		
MDD	4	–
Social anxiety	1	–
Psychosis	1	–
Eating disorder	1	–
Hypertension	1	–
Restless legs	1	–
Medication		
Methyphenidate	9	–
Bupropion//Sertraline/Venlafaxine/Paroxetin	4	–
Risperidon	1	
Adult sensory profile		
Low registration**	38.61 (8.8)	24.94 (6.5)
Sensory avoidance**	42.39 (9.3)	35.31 (6.9)
Sensation seeking	47.72 (8.1)	47.00 (6.3)
Sensory sensitivity**	42.78 (8.5)	33.06 (7.1)

*SD* standard deviation, *CAARS* Conners Adult ADHD Rating Scales, *Hyp./Imp.* hyperactivity/impulsivity, *Wursk* Wender-Utah Rating Scale, *BDI* Beck Depression Inventory, *BSL* borderline symptom list, *MDD* major depressive disorder

*p < 0.05; **p < 0.001 calculated with independent sample *t* test

### Perceptual load

#### Sensory conditions

**Fig. 1:**
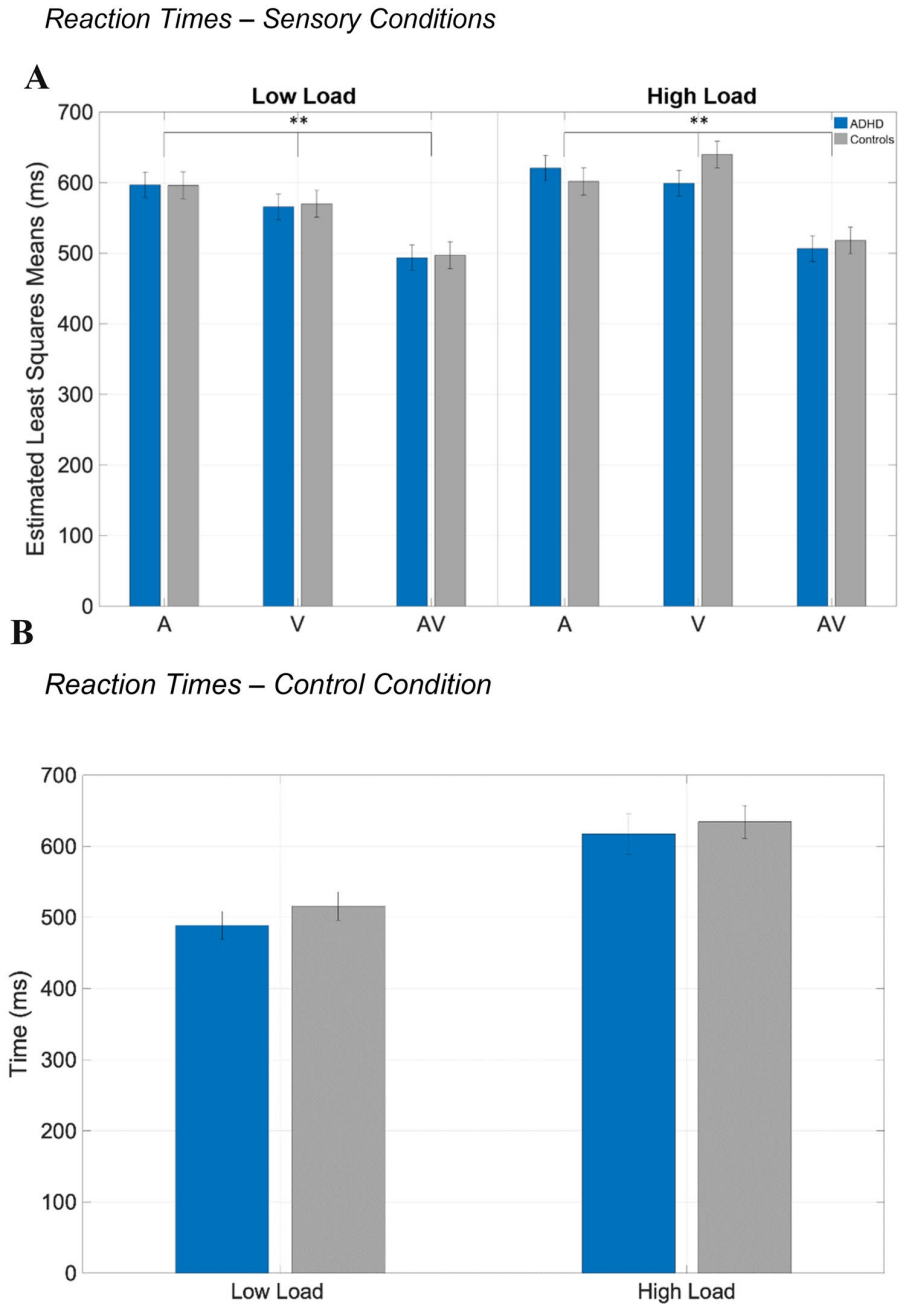
**A** Predicted reaction times for sensory conditions; *A* auditory, *V* visual, *AV* audiovisual; **B** reaction times of the control-condition

LMM with fixed factors of group, loading and modality showed no group differences of reaction time (*χ*^2^ = 0.068, *p* = 0.79). However, a significant group*loading*modality interaction was found (*χ*^2^ = 13.62, *p* < 0.001). This effect is explained by faster reaction time estimated least square means for multimodal stimuli, compared to unimodal stimuli (see Fig. 1) suggesting successful MI for both groups. MI within-group comparisons for load revealed no significant differences (*p*_ADHD_ = 1.0; *p*_controls_ = 0.3). In terms of errors, ANOVA revealed no group differences for commission errors (*F*(1, 35) = 2.251, *p* = 0.13) and commission errors (*F*(1,35) = 0.38, *p* = 0.68).

#### Control condition

ANOVA analysis revealed a significant effect for reaction times for load (*F*(1,35) = 154.4, *p* < 0.001) but not for group (*F*(1,35) = 0.26, *p* = 0.61). Similarly, commission errors were significant for load (*F*(1,35) = 8.93, *p* = 0.005) but not for groups (*F*(1,35) = 0.76, *p* = 0.38). Load led also to more omission errors (*F*(1,35) = 29,86, *p* < 0.001) but not different across groups (*F*(1,35) = 0.17, *p* = 0.6).

### Race model

**Fig. 2:**
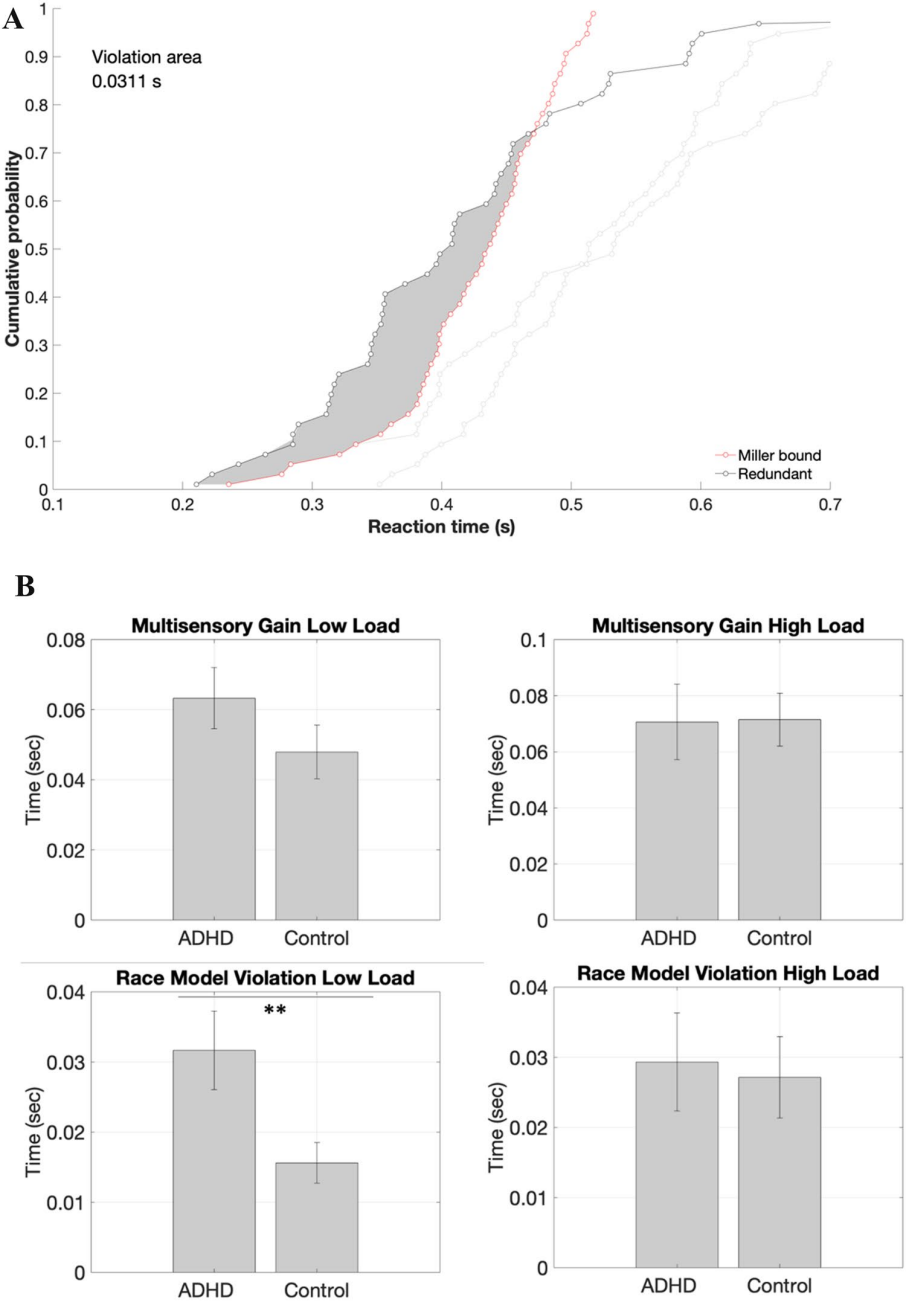
**A** Sample race model violation plot; **B** group-wise comparison of multisensory gain and race model violation; **p < 0.001

One sample *t* tests between empirical and predicted CDF revealed significantly race model violations for low load for ADHD patients (*t*(17) = 0.08, *p* < 0.001) and controls (*t*(17) = 0.03, *p* = 0.004). For high load, only controls showed race model inequalities (*t*(17) = 0.02, *p* = 0.006), but not ADHD patients (*t*(17) = − 0.02, *p* = 0.21). No group differences were evident for multisensory benefit (see Fig. 2). ADHD patients violated the race model significantly higher in the low-load condition compared to controls (*t*(35) = 0.002, *p* = 0.019), whereas there were no differences for the high-load condition (*t*(35) = − 0.016, *p* = 0.81).

## Discussion

This is the first study systematically addressing the robustness of MI in an adult ADHD sample for simple stimuli with varying perceptual loads. We hypothesized that ADHD patients cannot successfully integrate when perceptual capacities are high. As a result, patients with ADHD showed robust integration rates similar to controls, irrespective of load. This was marked by faster reaction times for multimodal stimuli compared to unimodal stimuli. Also, error rates across all conditions were similar to healthy controls, further suggesting that MI is not load-dependent in adult ADHD.

The load manipulation was successful, since we found significant higher reaction times in the high-load condition compared to low load for both groups in the visual search task. However, load manipulation did not have an influence on attentional capture to the sensory stimuli. This mirrors the ongoing debate of the role of attention and MI. On the one hand, stimuli can be integrated bottom–up driven via a pre-attentive stage, without the need of attentional deployment from higher-order areas [8, 9]. This type of integration is facilitated through stimulus features, such as temporal and spatial coincidence, with the closer they occur, the more likely MI takes place pre-attentively. On the other hand, more complex stimuli (e.g., linguistic material) require top–down attentional deployment. In our study, the applied stimuli comprised low-level properties as we used simple beeps and flashes. Therefore, it could be the case that in our study, the automaticity of stimuli processing is independent of the load since the basal stimuli may be integrated at a pre-attentive stage. The goal of the study was to challenge the robustness of MI for simple stimuli in ADHD. Consequently, one can conclude, that the process of bottom–up MI seems to be robust in ADHD and is not attenuated by additional task demands. To further challenge the robustness of load-dependent bottom–up MI, it would be advisable to manipulate the stimulus complexity. It has been shown, that MI for complex, speech-like stimuli, that require top–down attention is mitigated in patients with ADHD associated with higher resting-state connectivity from polymodal to unimodal areas [12]. A study by Alsius et al. could show, when a dual task demands high levels of attention, MI for speech-like stimuli declines [35]. It would be intriguing to study that phenomenon in ADHD as to whether MI for complex further mitigates in a dual task compared to simple task. Interestingly, the phenomenon of declining MI performance for complex stimuli in dual tasks could be explained by a narrowed temporal window of integration (TWI), i.e., maximum temporal characteristics between sensory events that allows their perceptual binding [36]. It has been hypothesized that, depending on the task demand, adaptively widening/narrowing of the TWI could take place, responsible for integration for simple stimuli (wide TWI) or declining integration (narrowed TWI). Abnormal TWI were associated with ADHD traits [37]. Further, in a temporal order judgment task, where participants were required to reproduce the sequence of heard tones, ADHD is associated with higher thresholds compared to healthy controls indicating poorer performance when two sensory events occur close in time [38]. To further challenge the robustness of MI in dual tasks for patients with ADHD, it would be desirable to alter the stimulus-onset asynchronies between the sensory events to investigate the role of TWI in ADHD closer.

In the current study, we found significant race model violations in ADHD evident in the low-load condition, but not in the high-load condition. The theoretical underpinning of the race model assumes that perceptual decision-making is a result of two ‘racing’ sensory modalities: when one sensory modality reaches a threshold of stimulation, it triggers a response, hence both sensory modalities are independent of each other. In contrast, the pooled approach assumes that two sensory modalities can be combined to reach a single supra-modal formation process [30, 39–42]. Since ADHD patients, did not violate the race model for the high-load condition, it could be that perceptual decision-making is altered in attentional demanding situations towards independent sensory accumulation. Additionally, we found that ADHD is associated with significant higher gaining from two sensory modalities in the low-load condition, which cautiously can be interpreted as reaching a supra-modal threshold faster compared to healthy controls. This in turn could be the result of sensory hypersensitivity as reported for ADHD and evident in significant higher ASP scores in the current study. It is also known, that dopamine plays a role in sensory evidence accumulation, more specifically at modulatory stages differentiating signal from noise [43]. Suboptimal dopamine levels have shown to lead to ineffective sensory accumulation [43, 44]. Since ADHD is a neurodevelopmental disorder especially with abnormalities in the dopaminergic pathways, it could be that perceptual decision-making is altered due to suboptimal dopamine levels. Altered perceptual decision-making must not necessarily result in a behavioural difference. Kwasa et al. also found no behavioural differences between ADHD patients and healthy controls, however, the neuronal signature differed [45]. In an auditory task, that required attentional deployment, weaker neuronal modulation was reported in networks involved in both bottom–up and top–down processing [45–48]. Further studies are necessary to systematically address a possible altered perceptual decision-making in adult ADHD.

### Limitations

Since autism spectrum disorders (ASD) are also associated with difficulties in sensory processing and MI [49, 50] and is comorbid to ADHD [51], we should have screened for ASD. However, we recruited the patients sample via the outpatient department where they got a proper diagnosis along with documentation of all comorbidities. Nevertheless, we cannot rule out the presence of ASD in our sample. Further, the existence of comorbidities, e.g., MDD could have also influenced our results. While we asked for discontinuation of the ADHD-related medication, some participants also took antidepressants or risperidone. Therefore, those medication could have influenced the behavioral performance.

## Conclusion

Bottom–up MI for adult ADHD is robust against additional task demands. The underlying sensory accumulation may differ compared to healthy controls but nevertheless leading to similar MI rates for simple stimuli. Future studies are needed to investigate the underlying perceptual decision-making and its possible limits for MI in adult ADHD more specifically.

## References

[1] Willcutt EG, Nigg JT, Pennington BF (2012). Validity of DSM-IV attention deficit/hyperactivity disorder symptom dimensions and subtypes. J Abnorm Psychol.

[2] Sibley MH, Mitchell JT, Becker SP (2016). Method of adult diagnosis influences estimated persistence of childhood ADHD: a systematic review of longitudinal studies. Lancet Psychiatry.

[3] Barkley RA, Edwards G, Laneri M (2001). Executive functioning, temporal discounting, and sense of time in adolescents with attention deficit hyperactivity disorder (ADHD) and oppositional defiant disorder (ODD). J Abnorm Child Psychol.

[4] Ghanizadeh A (2011). Sensory processing problems in children with ADHD, a systematic review. Psychiatry Investig.

[5] Bijlenga D, Tjon-Ka-Jie JYM, Schuijers F, Kooij JJS (2017). Atypical sensory profiles as core features of adult ADHD, irrespective of autistic symptoms. Eur Psychiatry.

[6] Panagiotidi M, Overton PG, Stafford T (2018). The relationship between ADHD traits and sensory sensitivity in the general population. Compr Psychiatry.

[7] Dhamala M, Assisi CG, Jirsa VK (2007). Multisensory integration for timing engages different brain networks. Neuroimage.

[8] MacAluso E, Noppeney U, Talsma D (2016). The curious incident of attention in multisensory integration: bottom–up vs top–down. Multisens Res.

[9] Talsma D (2015) Predictive coding and multisensory integration: an attentional account of the multisensory mind. Front Integr Neurosci 10.3389/fnint.2015.00019 PMC4374459 25859192

[10] Michalek AMP, Watson SM, Ash I (2014). Effects of noise and audiovisual cues on speech processing in adults with and without ADHD. Int J Audiol.

[11] McCracken HS, Murphy BA, Glazebrook CM et al (2019) Audiovisual multisensory integration and evoked potentials in young adults with and without attention-deficit/hyperactivity disorder. Front Hum Neurosci 13 10.3389/fnhum.2019.00095 PMC6433696 30941026

[12] Schulze M, Aslan B, Stöcker T (2021). Disentangling early versus late audiovisual integration in adult ADHD: a combined behavioural and resting-state connectivity study. JPN.

[13] Huang-Pollock CL, Carr TH, Nigg JT (2002). Development of selective attention: perceptual load influences early versus late attentional selection in children and adults. Dev Psychol.

[14] Portas C, Rees G, AH-J of, 1998 undefined (1998). A specific role for the thalamus in mediating the interaction of attention and arousal in humans. Soc Neurosci.

[15] Lavie N (1995). Perceptual load as a necessary condition for selective attention. J Exp Psychol Hum Percept Perform.

[16] Lunn J, Sjoblom A, Ward J (2019). Multisensory enhancement of attention depends on whether you are already paying attention. Cognition.

[17] Santangelo V, Spence C (2007). Multisensory cues capture spatial attention regardless of perceptual load. J Exp Psychol Hum Percept Perform.

[18] Santangelo V, Botta F, Lupiáñez J, Spence C (2010). The time course of attentional capture under dual-task conditions. Attent Percep Psychophys.

[19] Forster S (2013). Plugging the attention deficit: perceptual load counters increased distraction in ADHD. Neuropsychology.

[20] Carreiro LR, Machado-Pinheiro W, Junior AA (2021). Adults with adhd symptoms express a better inhibitory capacity when the perceptual load is higher. Eur Psychiatry.

[21] NICE (2018) Attention deficit hyperactivity disorder: diagnosis and management (NICE Guideline) 29634174

[22] Heinzl S (2018). Neue S3-Leitlinie ADHS bei Kindern, Jugendlichen und Erwachsenen. DNP Der Neurologe & Psychiater.

[23] Christiansen H, Kis B, Hirsch O (2012). German validation of the Conners Adult ADHD Rating Scales (CAARS) II: reliability, validity, diagnostic sensitivity and specificity. Eur Psychiatry.

[24] Retz-Junginger P, Retz W, Blocher D (2002). Wender Utah rating scale (WURS-k): Die deutsche kurzform zur retrospektiven erfassung des hyperkinetischen syndroms bei erwachsenen. Nervenarzt.

[25] Bohus M, Kleindienst N, Limberger MF (2009). The short version of the Borderline Symptom List (BSL-23): development and initial data on psychometric properties. Psychopathology.

[26] Beck AT, Steer RA, Brown GK (1996). Manual for the Beck depression inventory-II.

[27] Brown C, Tollefson N, Dunn W (2001). The adult sensory profile: measuring patterns of sensory processing. Am J Occup Ther.

[28] Bates D, Mächler M, Bolker B, Walker S (2014) Fitting linear mixed-effects models using lme4. J Stat Softw 67

[29] Boisgontier MP, Cheval B (2016). The anova to mixed model transition. Neurosci Biobehav Rev.

[30] Otto TU, Mamassian P (2012). Noise and correlations in parallel perceptual decision making. Curr Biol.

[31] Innes BR, Otto TU (2019). A comparative analysis of response times shows that multisensory benefits and interactions are not equivalent. Sci Rep.

[32] Colonius H, Diederich A (2020). Formal models and quantitative measures of multisensory integration: a selective overview. Eur J Neurosci.

[33] Otto TU (2019). RSE-box: an analysis and modelling package to study response times to multiple signals. Quant Methods Psychol.

[34] Colonius H, Diederich A (2006). The race model inequality: interpreting a geometric measure of the amount of violation. Psychol Rev.

[35] Alsius A, Navarra J, Campbell R, Soto-Faraco S (2005). Audiovisual integration of speech falters under high attention demands. Curr Biol.

[36] Michail G, Keil J (2018). High cognitive load enhances the susceptibility to non-speech audiovisual illusions. Sci Rep.

[37] Panagiotidi M, Overton PG, Stafford T (2017). Multisensory integration and ADHD-like traits: evidence for an abnormal temporal integration window in ADHD. Acta Physiol (Oxf).

[38] Fostick L (2017). The effect of attention-deficit/hyperactivity disorder and methylphenidate treatment on the adult auditory temporal order judgment threshold. J Speech Lang Hear Res.

[39] Mercier MR, Cappe C (2020). The interplay between multisensory integration and perceptual decision making. Neuroimage.

[40] Bizley JK, Jones GP, Town SM (2016). Where are multisensory signals combined for perceptual decision-making?. Curr Opin Neurobiol.

[41] Bogacz R (2007). Optimal decision-making theories: linking neurobiology with behaviour. Trends Cogn Sci.

[42] Smith PL, Ratcliff R (2004). Psychology and neurobiology of simple decisions. Trends Neurosci.

[43] Beste C, Adelhöfer N, Gohil K (2018). Dopamine modulates the efficiency of sensory evidence accumulation during perceptual decision making. Int J Neuropsychopharmacol.

[44] Vijayraghavan S, Wang M, Birnbaum SG (2007). Inverted-U dopamine D1 receptor actions on prefrontal neurons engaged in working memory. Nat Neurosci.

[45] Kwasa JAC, Noyce AL, Torres LM, Shinn-Cunningham BG (2021) Main Manuscript for top–down attention modulates auditory-evoked neural responses in neurotypical, but not ADHD, young adults. 10.1101/2021.02.11.430824

[46] Hasler R, Perroud N, Meziane HB (2016). Attention-related EEG markers in adult ADHD. Neuropsychologia.

[47] Barry RJ, Clarke AR, McCarthy R (2009). Event-related potentials in adults with Attention-Deficit/Hyperactivity Disorder: an investigation using an inter-modal auditory/visual oddball task. Int J Psychophysiol.

[48] Salmi J, Salmela V, Salo E (2018). Out of focus—brain attention control deficits in adult ADHD. Brain Res.

[49] Stevenson RA, Siemann JK, Woynaroski TG (2014). Evidence for diminished multisensory integration in autism spectrum disorders. J Autism Dev Disord.

[50] van Laarhoven T, Stekelenburg JJ, Vroomen J (2019). Increased sub-clinical levels of autistic traits are associated with reduced multisensory integration of audiovisual speech. Sci Rep.

[51] Gargaro BA, Rinehart NJ, Bradshaw JL et al (2011) Autism and ADHD: How far have we come in the comorbidity debate? Neurosci Biobehav Rev 10.1016/j.neubiorev.2010.11.002 21093480

